# Disparate Phenotypes Resulting from Mutations of a Single Histidine in Switch II of *Geobacillus stearothermophilus* Translation Initiation Factor IF2

**DOI:** 10.3390/ijms21030735

**Published:** 2020-01-22

**Authors:** Jerneja Tomsic, Arianna Smorlesi, Enrico Caserta, Anna Maria Giuliodori, Cynthia L. Pon, Claudio O. Gualerzi

**Affiliations:** Laboratory of Genetics, Department of Biosciences and Biotechnology, University of Camerino, 62032 Camerino, Italycynthia.pon@unicam.it (C.L.P.)

**Keywords:** protein synthesis, translation initiation, IF2 structure, switch II mutations, IF2 recycling, GTP hydrolysis

## Abstract

The conserved Histidine 301 in switch II of *Geobacillus stearothermophilus* IF2 G2 domain was substituted with Ser, Gln, Arg, Leu and Tyr to generate mutants displaying different phenotypes. Overexpression of IF2H301S, IF2H301L and IF2H301Y in cells expressing wtIF2, unlike IF2H301Q and IF2H301R, caused a dominant lethal phenotype, inhibiting in vivo translation and drastically reducing cell viability. All mutants bound GTP but, except for IF2H301Q, were inactive in ribosome-dependent GTPase for different reasons. All mutants promoted 30S initiation complex (30S IC) formation with wild type (wt) efficiency but upon 30S IC association with the 50S subunit, the fMet-tRNA reacted with puromycin to different extents depending upon the IF2 mutant present in the complex (wtIF2 ≥ to IF2H301Q > IF2H301R >>> IF2H301S, IF2H301L and IF2H301Y) whereas only fMet-tRNA 30S-bound with IF2H301Q retained some ability to form initiation dipeptide fMet-Phe. Unlike wtIF2, all mutants, regardless of their ability to hydrolyze GTP, displayed higher affinity for the ribosome and failed to dissociate from the ribosomes upon 50S docking to 30S IC. We conclude that different amino acids substitutions of His301 cause different structural alterations of the factor, resulting in disparate phenotypes with no direct correlation existing between GTPase inactivation and IF2 failure to dissociate from ribosomes.

## 1. Introduction

Translation initiation factor IF2 is a multi-domain GTPase playing essential roles in the initiation pathway of protein synthesis (for reviews see [1,2,3,4,5,6,7,8,9]). IF2 binds with high affinity to the 30S ribosomal subunit with its N-terminal domain [9,10,11,12,13,14] and subsequently establishes a functional interaction with the ribosome [15,16], mainly through its G2 and G3 domains [12,13,17,18,19,20,21,22]. 30S-bound IF2 recruits the initiator fMet-tRNA [23] which is held in place on the subunit by an interaction between its acceptor end and the C-terminal domain C-2 of the factor [24,25,26] and by pairing of the anticodon bases with the mRNA initiation codon at a “pre-P-site” of the subunit [9,18,19,20,21,27]. During these early steps, a GTP molecule is bound to the G2 domain [17,18,28,29] conferring upon IF2 a “GTP conformation” [18,19,20,21,22,29,30,31]. In this conformation, the affinity for the 30S subunit is higher than in the presence of GDP, ppGpp or in the absence of guanosine nucleotides [28,32,33,34,35,36] and the docking of the formed 30S initiation complex (30S IC) with the 50S subunit is faster [35,36]. IF2-bound GTP is immediately hydrolyzed upon joining the 50S with the 30S IC [35,36,37,38,39] and GDP-Pi remains IF2- and ribosome-bound for about 200 msec, until the Pi is dissociated [9,37,38]. IF2 eventually promotes the adjustment of the acceptor end of initiator fMet-tRNA in the productive P site of the 50S subunit so that the initiation dipeptide can be formed with the amino acid carried to the A site by an aminoacyl-tRNA•EF-Tu•GTP ternary complex [32,37,38,39].

The function of the GTP hydrolysis upon formation of the 70S IC has been the subject of considerable controversy. According to one hypothesis, the energy generated by the hydrolysis is necessary to promote a conformational change of the factor, which in turn would allow its dissociation from the ribosome [35,40,41,42]. However, another study demonstrated that a single amino acid substitution (E571K) in the G3 domain of *Escherichia coli* IF2 resulted in a mutant factor which, although completely inactive in GTP hydrolysis, was capable of performing in vitro all translation initiation functions of wild type (wt) IF2 and was able to replace wtIF2 in vivo allowing the cells to grow with almost wt duplication times [34]. The conclusion drawn from this study was that the energy generated by GTP hydrolysis is not necessary to allow IF2 dissociation but that the hydrolysis is required to remove the γ-phosphate of GTP which is toxic insofar as its presence prevents a conformational change required to allow IF2 recycling [34,37,38].

**Figure 1:**
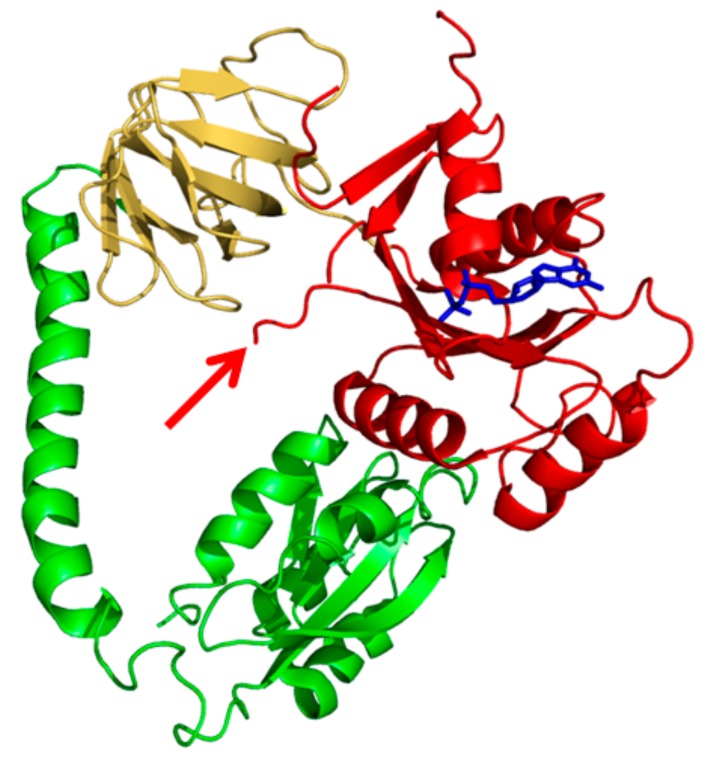
3D structure of of *Thermus thermophilus* IF2 bound to GDP (blue). The IF2 domains are colour-coded: G2 (red), G3 (yellow) and C1 (green). The red arrow indicates the position of His301 at the tip of switch II. The figure is from pdb file 4KJZ.

Histidine 301 of *Geobacillus* (formerly *Bacillus) stearothermophilus* IF2 is situated at the edge of the consensus sequence (DTPGH) of switch II (Figure 1), a well conserved structural element present in initiation and elongation factors of different organisms. Switch II changes its structure depending upon the nature of the guanine nucleotide present and is involved in functionally significant conformational changes of the factor [43,44]. When His84 (homologous to His301 of *G. stearothermophilus* and to His448 of *E. coli* IF2) was substituted with Gly in the G-domain of *E. coli* EF-Tu, the GTPase activity of the elongation factor was reduced by ca. 95% despite the fact that the interaction of the factor with GDP and GTP remained unaffected, indicating that this residue is not directly involved in binding of the GTP γ-phosphate [45]. 

Two equivalent mutations introduced in His448 of *E. coli* IF2 generated proteins which had lost their GTPase activity and failed to be released from the ribosomes upon formation of the 70S IC [42]. This finding led the authors to conclude that GTP hydrolysis is necessary for IF2 recycling off the ribosome. 

In light of the fact that this is the main finding which purports to establish a direct cause-effect relationship between IF2-dependent GTP hydrolysis and IF2 recycling off the ribosomes in the present work we have re-examined properties and phenotypes of several *G. stearothermophilus* IF2 mutants carrying substitutions of the His301 residue (equivalent to H448 of *E. coli* IF2). 

Our data confirm that, whereas all the mutants are indeed defective in IF2 dissociation upon 70S IC formation, not all the mutants are defective in GTP hydrolysis The present data allow us to conclude that the failure of IF2 to dissociate, like other disparate functional defects displayed by these mutants, cannot be attributed to the inactivation of the GTPase but are instead due to mutation-specific alterations of the IF2 structure.

## 2. Results

### 2.1. Effect of the In Vivo Expression of IF2H301 Substitution Mutants

**Figure 2:**
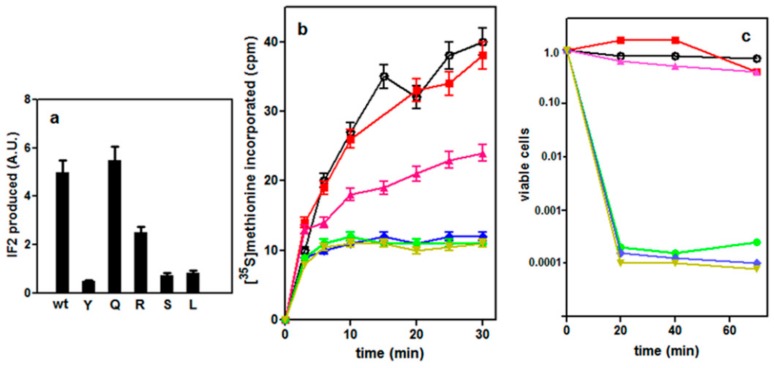
Dominant lethal phenotype caused by overproduction of IF2H301 substitution mutants. (**a**) Amounts of wtIF2 and IF2His301 mutants (as indicated in the abscissa) detected in *E. coli* cells 30 min after induction of their expression; (**b**) in vivo [^35^S] methionine incorporation in acid insoluble material as a function of the times (indicated in the abscissa) after induction of expression of the IF2 variants; (**c**) viable *E. coli* cells counted after induction of the IF2 variants. The symbols in panels (b) and (c) correspond to: wtIF2 (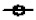); IF2H301Y(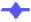); IF2H301Q(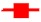); IF2H301L(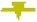); IF2H301R (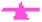) and IF2H301S(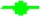) expression. Further details are given in Materials and Methods.

Residue H301 of *G. stearothermophilus* IF2 was substituted with five different amino acids (Tyr, Ser, Leu, Gln and Arg) having different chemical properties. The *infB* mutants thus obtained were cloned in pPLc*2833*, the plasmid already used to express wtIF2. The newly generated plasmids (see Materials and Methods in the Supplementary Materials) were transformed into *E. coli* UT5600 which contains a normal chromosomal copy of *infB* and harbors pcI*857* expressing a ts mutant of bacteriophage λ repressor. SDS-PAGE analyses of samples taken from these cells before and after induction of IF2 overexpression revealed that only IF2H301Q and IF2H301R were expressed at fairly high levels, whereas very little expression of the other mutants (i.e., IF2H301Y, IF2H301S and IF2H301L) could be detected 30 min after induction (Figure 2a). The failure to express these mutant proteins was accompanied by a substantial decrease of the synthesis of the bulk cellular proteins, as seen from the result of the pulse-chase experiment in which the incorporation of [^35^S] methionine was compared in cells induced to express either wtIF2 or His301 mutants. The results obtained upon expression of IF2H301Y are shown in Figure S1.

These results were interpreted as resulting from a dominant lethal toxic effect of some of these His301 mutants. That this is indeed the case is shown by the finding that protein synthesis in vivo is severely inhibited upon induction of IF2H301S, IF2H301L and IF2H301Y expression whereas induction of IF2H301Q had no negative effect and expression of IF2H301R produced a clear reduction of protein synthesis compared to wild-type, but failed to inhibit completely translation (Figure 2b). 

The dominant lethal phenotype is further confirmed by the finding that the number of viable cells in the culture decreased by at least 4 orders of magnitude upon induction of IF2H301S, IF2H301L and IF2H301Y expression, whereas no loss of viability ensued the expression of wtIF2, IF2H301Q, and IF2H301R (Figure 2c). The target of the toxicity caused by the IF2 mutants was investigated in an experiment in which the incorporation of precursors of peptidoglycan (Figure S2a), DNA (Figure S2b), RNA (Figure S2c) and protein (Figure S2d) syntheses was followed in cells which were induced to overexpress wtIF2 or the toxic His301 IF2 mutants. Because methionine is the only precursor whose incorporation is inhibited, the results clearly indicate that the toxicity entails an exclusive inhibition of protein synthesis (Figure S2d). 

To understand how protein synthesis is blocked in the presence of some IF2H301 mutants, it was necessary to carry out in vitro studies using purified mutated IF2 molecules. However, whereas the proteins which had no negative effect on cell viability (i.e., wtIF2, IF2H301Q and IF2H301R) were purified after hyperexpression in *E. coli*, it was impossible to obtain a reasonable amount of the toxic mutants (i.e., IF2H301Y, IF2H301L and IF2H301S) in *E. coli.* Therefore, these proteins were purified after heterologous hyperexpression in *Saccharomyces cerevisiae* exploiting the known differences existing between bacterial and eukaryotic translation initiation.

### 2.2. Ribosome-dependent GTPase Activity of the IF2 Mutants

**Figure 3:**
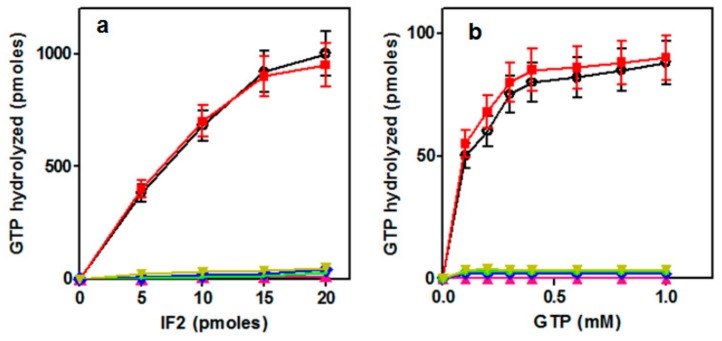
Ribosome-dependent GTP hydrolysis by IF2. (**a**) Amount (pmoles) of GTP hydrolyzed after 20 min incubation at 45 °C of 100 μM GTP with the increasing amounts (abscissa) of the individual IF2 variants in the presence of *G. stearothermophilus* 30S and 50S ribosomal subuniuts (15 pmoles each); (**b**) amount (pmoles) of GTP hydrolyzed by one pmole of IF2 after incubation of 10 pmoles of the individual IF2 variants under the same conditions described for panel (a). The symbols in both panels correspond to: wtIF2 (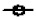); IF2H301Y(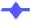); IF2H301Q(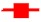); IF2H301L(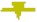); IF2H301R (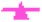) and IF2H301S(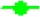).

To determine whether the observed protein synthesis inhibitions are connected with a diminished GTPase activity of the IF2His301 mutants, ribosome-dependent GTPase activity was tested in the presence of increasing amounts of either purified IF2 mutants (Figure 3a) or GTP (Figure 3b). The results of these experiments demonstrate that, like wtIF2, only IF2His301Q was able to efficiently hydrolyze GTP; all the other mutants were completely defective in this function under all experimental conditions tested. It is remarkable that IF2His301R, which does not confer a dominant lethal phenotype and does not inhibit protein synthesis, is completely inactive in GTP hydrolysis. From these findings it can be concluded that loss of GTPase activity, inhibition of translation and cell toxicity do not represent interrelated properties of this mutated factor.

### 2.3. The Reason for Loss of GTPase Activity Is Different in Different IF2His301 Mutants

The following experiments were carried out with the aim of investigating the reason for the inactivation of the GTPase following His301 substitution with Tyr, Leu, Ser and Arg. 

#### 2.3.1. GTP Binding to the IF2H301 mutants 

A trivial explanation for the loss of GTPase activity could be an inability to bind the guanosine nucleotide. Therefore the affinity for GTP of the mutant factors was preliminarily measured using a fluorescent GTP derivative (mant-GTP) which, as previously shown in the case of EF-G [46], undergoes an increase of the fluorescence emission upon binding to IF2. A fluorescence increase of mant-GTP, similar to that seen with wtIF2, was observed with all the IF2 mutants, indicating that binding of the nucleotide occurs in all cases. Subsequent titration experiments revealed that the K_d_’s of all mant-GTP complexes formed by the IF2 mutants were approximately two fold higher than that of wtIF2, indicating that the His 301 mutations cause only a slight reduction of the binding affinity for GTP (Table 1). Thus, in light of the near-normal binding of GTP, it can be concluded that a defect in GTP binding cannot explain the loss of GTPase activity by the IF2H301 mutants.

Alternative explanations for the failure of the mutants to hydrolyze GTP could be an inactivation of their catalytic center, which is an intrinsic feature of the G2 domain of the factor, and/or a defect in their interaction with the 50S ribosomal subunits which is responsible for the activation of the catalytic center. The following experiments were carried out to test these hypotheses. 

#### 2.3.2. Ribosome-Independent GTPase Activity of IF2

**Figure 4:**
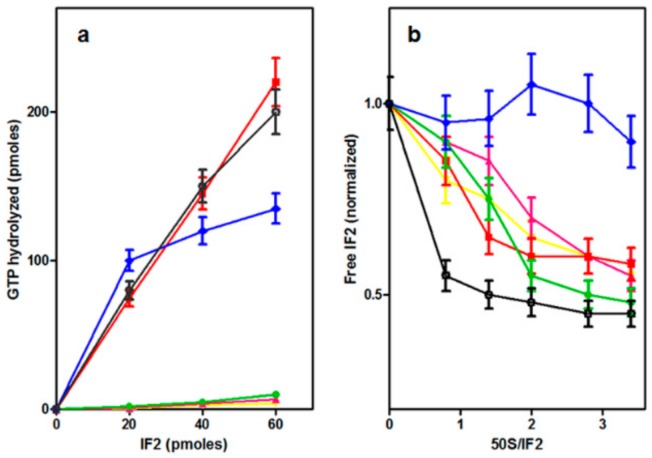
Ribosome-independent GTP hydrolysis by IF2. (**a**) GTP hydrolyzed (ordinate) in the presence of increasing amounts (abscissa) of the individual IF2 variants. The reaction mixtures and conditions were identical to those described in the legend to Figure 3 but for the fact that the ribosomal subunits were omitted and replaced by 20% ethanol; (**b**) interaction between increasing amounts of *G. stearothermophilus* 50S ribosomal subunits and a fixed amount of the individual IF2 variants so as to obtain the 50S/IF2 stoichiometric ratios indicated in the abscissa. After incubation for **10** min at 45 °C the mixtures were subjected to ultracentrifugation and the amount of free (not 50S-bound) IF2 present in the post-ribosomal supernatant determined as described in Materials and Methods. The results are normalized with respect to the amount of each IF2 variant present in the absence of 50S subunits which is taken as = 1. The symbols in both panels correspond to: wtIF2 (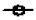); IF2H301Y(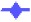); IF2H301Q(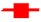); IF2H301L(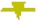); IF2H301R (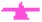) and IF2H301S(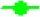).

It has been shown that exposure to low concentrations of aliphatic alcohols (e.g., 10–20% ethanol) of either intact IF2 [47] or of the isolated G2 domain [29] activates the catalytic center of the factor, resulting in ribosome-independent GTP hydrolysis. Thus, to measure of the intactness of the GTPase catalytic center of the various H301 mutants, ribosome-independent GTPase activity was tested. The results of this experiment show that GTP hydrolysis occurs with wtIF2, IF2H301Q and IF2H301Y, whereas it is absent in IF2H301R, IF2H301S and IF2H301L (Figure 4a).

#### 2.3.3. Binding of IF2H301 Mutants to 50S Subunits

In light of the above evidence that the GTPase center is still active in IF2H301Y, we sought to determine whether the latter mutant fails to hydrolyze GTP because of a defective interaction with the 50S subunits. For this purpose, the affinity of wtIF2 and of the various IF2 mutants for the large ribosomal subunit were compared in “pull down” experiments in which the factors were incubated with increasing amounts of 50S ribosomal subunits and subjected to ultracentrifugation. The amount of free IF2 (i.e., not bound to the 50S) present in the post-ribosomal supernatant was then determined. As seen from the results presented in Figure 4b, the IF2H301Y mutant is the only one which does not sediment with the 50S subunits for which it seems to have lost most of its affinity. On the other hand, increasing amounts of the other mutants were sedimenting upon centrifugation after incubation at increasing 50S/IF2 ratios, indicating that they are able to bind the 50S subunits, albeit with a somewhat reduced affinity compared to wtIF2. 

### 2.4. Activity of the IF2 Mutants in Supporting Single Steps of the Translation Initiation Pathway

#### 2.4.1. Binding of fMet-tRNA_f_^Met^ to Ribosomes

**Figure 5:**
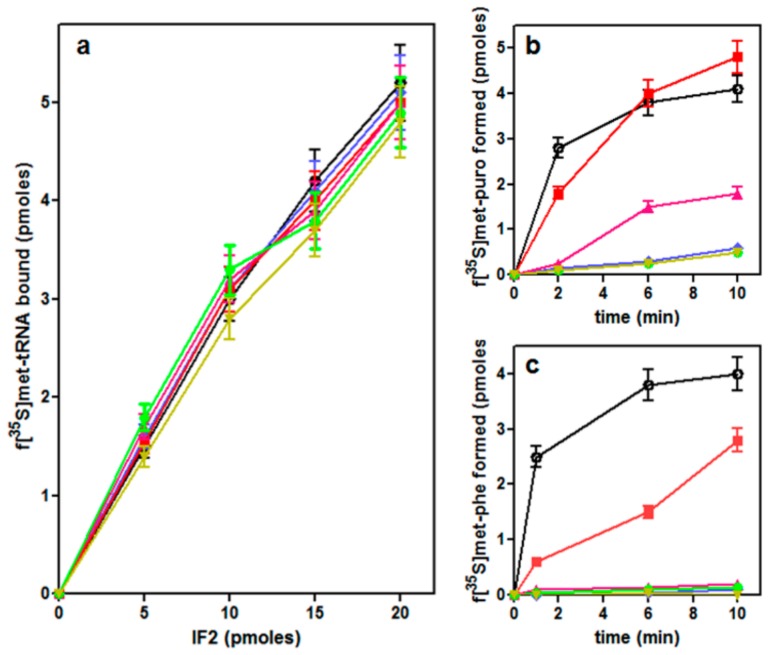
Activity of wtIF2 and IF2His301 mutants in performing individual functions of the translation initiation pathway. (**a**) Binding of fMet-tRNA to mRNA-programmed 30S ribosomal subunits to form a 30S IC in the presence of wtIF2 or mutant IF2; (**b**) time course of fMet-puromycin formation upon addition of 50S subunits and puromycin to 30S IC prepared in the presence of the different types of IF2; (**c**) time course of fMet-Phe initiation dipeptide formation upon addition of EF-Tu•GTP•Phe-tRNA ternary complexes to 70S initiation complexes assembled with 30S IC prepared in the presence of the different types of IF2. In all three panels the symbols correspond to: wtIF2 (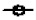); IF2H301Y(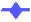); IF2H301Q(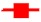); IF2H301L(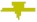); IF2H301R (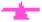) and IF2H301S(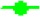). Further details are given in Materials and Methods.

A major role of IF2 is to promote fMet-tRNA^fMet^ binding to the 30S ribosomal subunits to form a 30S IC [23]. The ability of the mutant IF2 molecules to promote this translation initiation step was measured in the presence of mRNA, initiation factors IF1, IF3 and GTP. As seen in Figure 5a, the amount of ribosome-bound fMet-tRNA_f_^Met^ increased to the same extent as a function of increasing amounts of wtIF2 and of all IF2 mutants present in the reaction mixtures, indicating that wtIF2 and all IF2 mutants have the same capacity to promote 30S IC formation. 

#### 2.4.2. Puromycin Reaction of Ribosome-Bound fMet-tRNA 

The IF2-dependent positioning of fMet-tRNA_f_^Met^ in the correct ribosomal P site is essential for the formation of the first peptide bond which yields the initiation dipeptide. A standard test to determine if an aminoacyl-tRNA occupies the ribosomal P site consists in assessing its reactivity with puromycin. When formation of fMet-puromycin was measured on complexes assembled in the presence of increasing amounts of different IF2 variants, almost equal amounts of fMet-puromycin were obtained with wtIF2 and IF2H301Q, whereas IF2H301R (especially in the presence of an excess amount of the factor) yielded approximately half the amount of fMet-puromycin obtained with wtIF2. Finally, very low levels of fMet-puromycin were obtained with IF2H301S, IF2H301L and IF2H301Y (Figure 5b).

#### 2.4.3. Initiation Dipeptide Formation 

Quite different results were obtained when the initiation complexes (i.e., 70S IC) were challenged by the addition of an EF-Tu•GTP•Phe-tRNA ternary complex to produce the fMet-Phe dipeptide. Only IF2H301Q was found to have some activity in promoting initiation dipeptide formation, albeit at a substantially reduced rate compared to wtIF2. All complexes formed with the other IF2His301 mutants proved completely inactive in dipeptide formation, indicating that they carry the initiator fMet-tRNA bound in a non-productive position (Figure 5c).

Because the GTPase activity of IF2 is not required for positioning fMet-tRNA_f_^Met^ in the P-site [34,37,39], the inability or the reduced ability of the IF2 mutants to support fMet-puromycin and initiation dipeptide formation cannot be correlated with their residual GTPase activity. 

### 2.5. Dissociation of IF2 from the Ribosome

**Figure 6:**
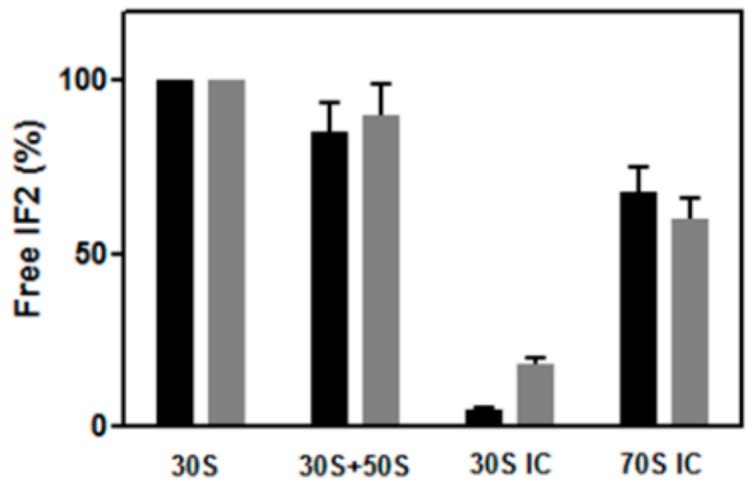
Dissociation of wtIF2 from the ribosomes. The histogram bars represent the amount of free (i.e., not ribosome-bound) wtIF2 present in the post-ribosomal supernatant after ultracentrifugation of samples containing 1 mM GTP (black bars) or 1 mM GDP (gray bars) and as indicated: wtIF2 and 30S ribosomal subunits; wtIF2, 30S and 50S ribosomal subunits; wtIF2 and all the components necessary for the formation of a 30S IC; wtIF2, 30S IC and 50S subunit forming a 70S IC. The amounts of free IF2 detected in the samples containing only 30S subunits are taken as equal to 100% and the amounts of free IF2 detected in the other samples are expressed as % of this amount. More experimental details are given in Materials and Methods.

To determine whether GTP hydrolysis is indeed essential for IF2 recycling off the ribosome, as suggested by the results obtained in *E. coli* with the GTPase-defective IF2 mutants of His448 [42], IF2 dissociation upon formation of 70S initiation complexes (70S IC) was assayed by “pull down” centrifugation experiments in which the amount of wtIF2 and IF2H301 mutants was measured in the post-ribosomal supernatants following ultracentrifugation of samples containing the IF2 variants and different combinations of ribosomal subunits and ribosomal ligands involved in translation initiation. As seen in Figure S3, upon ultracentrifugation following the incubation of wtIF2 with 30S ribosomal subunits, the bulk of IF2 remains unbound and is found it the post-ribosomal supernatant while only a very small portion of IF2 is ribosome-bound and sediments with the ribosomal particles. In the presentation of our results the amount of IF2 remaining free (i.e., unbound) upon incubation with vacant 30S subunits is taken as 100% and all the data have been normalized with respect to this amount. Incubation of IF2 with both 30S and 50S ribosomal subunits causes a small decrease of the amount of free IF2, indicating that slightly more IF2 is bound in the presence of both subunits. However, the amount of 30S-bound IF2 is substantially increased upon incubation with 30S subunits containing all the ribosomal ligands (i.e., mRNA, fMet-tRNA, IF1, IF3) involved in 30S IC formation. Under these conditions only a small percentage of IF2 remains free and is found in the supernatant, confirming the notion that the affinity of IF2 for the 30S ribosomal subunit is greatly increased upon 30S IC formation. Upon formation of 70S IC which follows the addition of 50S subunits to the 30S IC, a large percentage of the IF2 molecules which were bound in the 30S IC is released and becomes free in the post-ribosomal supernatant. Because the dissociation of IF2 upon 70S IC formation is extensive yet not complete, we tried to see if the reaction of fMet-tRNA with puromycin or formation of the initiation dipeptide upon addition of a EF-Tu⋅GTP⋅Phe-tRNA ternary complex would increase the level of dissociation; however, in neither case the amount of unbound IF2 increased, suggesting that a small proportion of IF2 bound to the 30S IC or 70S IC occupies a non-canonical position and remains associated with the ribosome. These results represent the proof of principle that pull-down experiments are suitable to determine the extent of IF2 dissociation upon 70S IC formation. Therefore, experiments similar to those just described were carried out in the presence of either GTP or GDP to determine if GTP (and its hydrolysis) can influence IF2 dissociation. As expected from what is known in the literature, the extent of IF2 binding to the vacant 30S subunits and to 30S IC was found to be somewhat (i.e., ~30%) lower in the presence of GDP compared to GTP [28,34]. Nevertheless, after normalization for these differences, the results clearly show that IF2 dissociation can occur also in the presence of GDP (Figure 6). However, because hydrolysis of IF2-bound GTP produces an IF2-GDP complex, these results do not rule out the possibility that GTP hydrolysis is needed to eject IF2 from the ribosome, but indicate only that IF2 dissociation can occur if the hydrolysis step is bypassed by complexing the factor with GDP.

**Figure 7:**
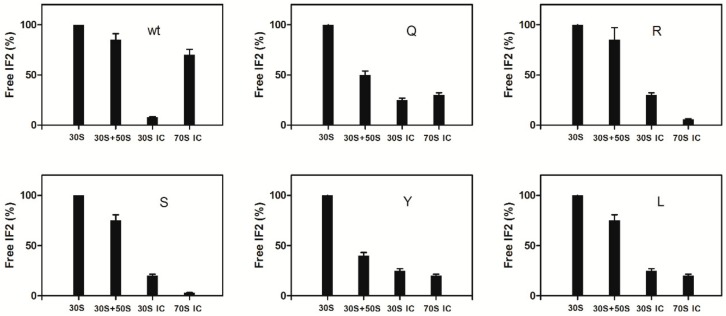
Failure of the IF2His301 mutants to dissociate from the ribosomes. The histogram bars represent the amount of free (i.e., not ribosome-bound) IF2 present in the post-ribosomal supernatant after ultracentrifugation. The experimental conditions are identical to those described for the experiment of Figure 6, but for the fact that all samples contained 1 mM GTP and different IF2His301 mutants as specified inside each panel. More experimental details are given in Materials and Methods.

The dissociation of the IF2His301 mutants was then analyzed in pull down experiments like those presented above. As seen from the results (Figure 7) the level of IF2 remaining free was essentially the same for all IF2 mutants after incubation with vacant 30S subunits but differed considerably for the different mutants upon incubation with both 30S and 50S subunits. 

In fact, whereas IF2H301R and H301S remained to a large extent unbound like wtIF2, both IF2H301Q and H301Y were ribosome-bound to a substantially higher level than wtIF2 indicating that these mutants have a stronger affinity than wtIF2 for the associated subunits (i.e., vacant 70S monomers). In light of the strongly reduced binding of the IF2H301Y to the isolated 50S subunit (Figure 4b) and of the normal affinity displayed for the 30S subunit, it would appear that this mutant is endowed with a strong affinity for the structure acquired by the subunits upon association. In agreement with the above finding that all the mutants promote 30S IC formation to the same extent, a large percentage of the IF2 molecules, similar in all variants yet somewhat lower than in the case of wtIF2, remained 30S IC-bound after ultracentrifugation. However, upon addition of 50S subunits to the 30S IC the IF2 mutants displayed a behavior drastically different from wtIF2. In fact, none of the mutant IF2s became free upon formation of the 70S IC unlike wtIF2 which was dissociated and became free. Instead of being dissociated, the amount of ribosome-bound IF2 increased, at least in the cases of IF2H301R and H301S, upon 70S IC formation (Figure 7).

## 3. Discussion

During the early events of translation initiation the IF2 activity is modulated by its interaction with guanine nucleotide ligands. IF2-GTP has a higher affinity than IF2-GDP for the 30S ribosomal subunit and is somewhat more efficient in promoting 30S IC formation in addition to signaling a favourable metabolic condition of the cell [28,33,34,48]. Furthermore, in the presence of IF2-GTP docking of the 50S ribosomal subunit to the 30S IC is faster [35,36,49]. Immediately upon association of the 50S subunits with the 30S IC, GTP is rapidly hydrolyzed, leaving GDP and inorganic Pi transiently bound to IF2. Subsequently, Pi is released from the complex and, concomitantly with the adjustment of fMet-tRNA in the P-site, IF2-GDP is eventually dissociated from the ribosomes [37,38,49]. 

The function of IF2-dependent GTP hydrolysis remained a controversial issue. The widespread belief that GTP hydrolysis is necessary to allow the recycling of IF2 off the ribosome is based on old observations that IF2 did not dissociate from the ribosomes when GTP was replaced by its non-hydrolyzable analogue GDPCP [50,51] and on the more recent findings that GTPase-defective mutants of IF2 (i.e., *E. coli* with either H448 or V400 substitution) failed to dissociate from the ribosomes [42]. 

However, the interpretation of these data does not take into account a number of experimental facts. First of all, the release of the γ-Pi of GTP is rate limiting for IF2 dissociation and for placing the initiator tRNA in the productive P-site, two strictly connected events. Thus, what is necessary to free IF2 is not the GTP hydrolysis itself but the removal of the toxic γ-Pi which blocks the final conformational change of IF2 which promotes the dissociation of the IF2 C2-fMet-tRNA interaction and in turn allows initiation dipeptide formation. In light of this it seems obvious that IF2 dissociation cannot occur in the presence of non-hydrolyzable GTP analogues because removal of the γ-Pi is not possible. Un-hydrolyzed GTP is advantageous during formation of 30S IC and the subsequent docking to the 50S subunit but its hydrolysis is only needed to get rid of the inhibitory γ-phosphate. Furthermore, the guanine nucleotide binding site of IF2 represents an ideal metabolic sensor devoted to gear translation initiation to the nutritional state of the cell. In fact, binding of GTP to IF2 is possible only under favourable conditions when its cellular concentration is high, whereas under starvation conditions the site is occupied by the alarmone ppGpp which inhibits the IF2 function thereby inhibiting protein synthesis [33].

That the energy of GTP hydrolysis is not required for the late events of the translation initiation pathway, including IF2 dissociation, is demonstrated by the recent evidence that all IF2 functions can be performed both in vitro and in vivo in the complete absence of GTP hydrolysis [34] and by an old finding that GTP can be removed by gel filtration from the 30S IC without impairing initiation dipeptide formation by the subsequently formed 70S IC [52]. In this connection, it should be stressed that IF2 and EF-Tu bind to overlapping ribosomal sites so that the dissociation or at least the displacement of IF2 from its ribosomal site is necessary to allow A-site binding of the aminoacyl-tRNA carried by the EF-Tu ternary complex. Unlike the late events of translation initiation, which do not seem to require IF2-dependent GTP hydrolysis, the cold-sensitive phenotype and the accumulation of ribosomal subunit precursors in the cells lacking the GTPase of IF2 indicate that this activity may play a role in assisting the protein chaperone activity of the factor during subunits assembly/maturation [53]. 

In the present article we have also shown that IF2•GDP can be dissociated during 70S IC formation, when GTP hydrolysis is bypassed. Most important, in this work the model of IF2 recycling based on the properties of the IF2His448 mutant has been challenged by the demonstration of the lack of correlation between the failure of IF2 to hydrolyze GTP and to dissociate upon 70S IC formation. This conclusion relies on the phenotypic properties displayed by different *G. stearothermophilus* IF2 variants carrying five single amino acid substitutions of His301 which corresponds to His448 of *E. coli* IF2. In fact, one of the mutants (IF2H301Q) preserved the capacity to hydrolyze GTP but, like the other mutants lacking the GTPase activity, failed to dissociate upon formation of a 70S IC. Furthermore, some of the IF2H301 mutants were found to inhibit protein synthesis in vivo and to cause a dominant lethal phenotype whereas other mutants did not display any toxic phenotype, despite being completely inactive in GTP hydrolysis. In three IF2 mutants the catalytic center proved to be inactive whereas in another case the GTPase defect could be traced back to a loss of affinity for the 50S subunit which is responsible for activating the hydrolytic activity. Thus, the cause of the defect in GTP hydrolysis was found to be different in the various mutants providing additional evidence that substitution of the same His residue with different amino acids results in disparate defects.

Overall, the results of this work demonstrate a complete lack of a cause-effect relationship between inactivation of the GTPase activity, dissociation of IF2 from the ribosomes and cell toxicity of the mutant protein. On the other hand, the five different His301 substitutions generated proteins with disparate phenotypes as far as capacity to hydrolyze GTP, affinity for the 70S ribosomes, capacity to allow the adjustment of initiator tRNA in the ribosomal P site so as to be puromycin reactive and to act as a donor in the formation of the first peptide bond. The disparate phenotypes displayed by the five His301 mutants (summarized in Table 2) lead us to conclude that any amino acid substitution in a critical position of the molecule such as switch II causes structural alterations which result in different types of defects, including the failure of IF2 to dissociate from the ribosome. Thus, the failure of IF2 to dissociate upon 70S IC formation cannot be attributed tout-court to the lack of GTPase activity but results instead from an altered structure of the factor. 

Furthermore, the model purporting GTPase-dependent dissociation of IF2 seemed to fit a functional model drawn from the conformational changes triggered by GTP hydrolysis in eIF5B, the archaeal/eukaryotic homologue of bacterial IF2 [54,55]. However, also the possible homology in the dynamic behavior upon GTP hydrolysis of IF2 and aIF5B based on the presumed structural homology between the two factors turned out to be incorrect. In fact, fitting a protein with the structural characteristics of eIF5B on a functional 30S IC or 70S IC appeared impossible from the very beginning [16,18,19,20,21] and more recent structural data obtained for IF2 have confirmed that the bacterial factor displays a different structural organization compared to eIF5B. In fact, NMR spectroscopy and X-ray diffraction studies [29,30,31] demonstrated that IF2 is unique among the translational GTPases because the effecter domains do not directly contact or form a stable interaction with the switch II region of the GTPase domain. IF2 displays a different structural organization compared to eIF5B IF2 and does not exhibit a chalice shape like eIF5B because switch II in the G2 domain of IF2 does not contact the C1 domain, precluding the possibility of communicating to the C2 domain the nature of the bound guanine nucleotide. This difference between IF2 and eIF5B is due to a difference in helix 8, which is more flexible and 24 residues longer in the bacterial factor. Furthermore, helix 12 of IF2, which connects domains C1 and C2, is not continuous and lacks the rigidity seen in eIF5B so that these domains display a completely independent mobility. As a consequence of these differences and of the different arrangement of their domains, eIF5B and IF2 do not use the same articulated lever mechanism to communicate to the fMet-tRNA binding domain C2 the structural signals generated by GTP hydrolysis. Thus, the IF2/eIF5B structural homology turns out to be misleading when used to construct functional models for IF2. 

When the 50S subunit joins the 30S IC, the interaction of IF2•GTP with domains II and VI of 23S rRNA and proteins L11 and L7/L12 of the 50S subunits [15,16,18,19,20,21,56,57,58] triggers GTP hydrolysis by the G2 domain of the factor which contains switch I, switch II and the P-loop. In turn, the G domain changes from the 30S IC to the 70S IC position causing the N-terminal domain to move into the solvent and lose some contacts with the ribosome thereby allowing IF2 to adopt a “ready-to-leave” conformation and to generate a 70S IC in which the ribosomal subunits are rotated with respect to each other [18,19,20,21]. In our opinion it is plausible that a structural alteration of the molecule which prevents in part or completely this conformational change is responsible for the disparate phenotypes displayed by the His301 substitution mutants.

## 4. Materials and Methods 

The following methods are described in Supplementary Materials: strains and growth media; site directed mutagenesis; cloning of *infB* mutants into pIM401 and pIM505; purification of *G. stearothermophilus* IF2; construction of the *S. cerevisiae* expression vector for *G. stearothermophilus* IF2 mutants; transformation of *S. cerevisiae* with the shuttle vector; Incorporation of thymine, uridine, histidine or N-acetylglucosamine.

### 4.1. General Preparations 

Ribosomal subunits (30S and 50S) of *G. stearothermophilus* and *E. coli* fMet-tRNA, initiation factors IF1 and IF3 and in vitro transcribed model 022 mRNA were prepared as described previously [59,60]. *E. coli* UT5600 harbouring different plasmids were grown to A_600_ ≅ 0.4 when the expression of IF2 (wt or mutants) was induced by shifting the cultures from 30 °C to 42 °C (taken as time 0). After 20 min at 42 °C the cultures were shifted back to 30 °C. Samples for the determination of viable cells were withdrawn at the indicated times.

### 4.2. Overexpression of the IF2 Variants 

*E. coli* UT5600 cells transformed with the plasmids encoding wild type *infB* or the mutated *infB* genes were incubated at 30 °C in LB supplemented with ampicillin and kanamycin. When the cultures reached A_600_ ≅ 0.8 the expression of the plasmid-encoded *infB* genes was induced by shifting the temperature to 42 °C for 15 min to inactivate the ts λP_L_ repressor. The cultures were then incubated at 37 °C to allow the expression of the *infB* genes. The level of each IF2 variant accumulated 30 min after induction of overexpression was estimated by SDS-PAGE analysis followed by silver staining and quantification by densitometry. In Figure 2a the protein levels are expressed in arbitrary units (A.U.).

### 4.3. Ribosome Dependent GTPase Activity of IF2 

A single incubation mixture contained wtIF2 or IF2H301 mutants (10 pmol), 30S and 50S subunits (15 pmol each), 100 µM [γ-^32^P], GTP (specific activity ~500 cpm/pmol) in 50 µL Buffer A (50 mM Tris-HCl, pH 7.5, 80 mM NH_4_Cl, 30 mM KCl, 7 mM MgCl_2_ and 1 mM DTT). The mixture was scaled up according to the number of samples to be taken during the time course of incubation at 37 °C.

### 4.4. Ribosome-Independent GTPase Activity of IF2 

Ethanol-stimulated GTP hydrolysis by IF2 was assayed as described [47]. The reaction mixtures in 50 µL Buffer A containing 20% ethanol were incubated at 37 °C for 2 h before being quenched by addition of 50 µL of quenching solution (1 M HClO_4_ and 3 mM KH_2_PO_4_). The amount of hydrolyzed [^32^P] γ-phosphate was determined by molybdate extraction as described [61].

### 4.5. Mant-GTP Binding to wtIF2 and IF2 Mutants 

Experiments were performed at 20 °C in Buffer A by rapidly mixing equal volumes (60 μL each) of IF2 (0.5 µM, concentration after mixing) with various concentrations (5 µM–40 µM) of mant-GTP and monitoring the time course of the fluorescence change. Upon excitation at 280 nm the single Trp residue (pos. 377) of *G. stearothermophilus* IF2 emits with maximum at 365 nm thereby exciting the mant-group of the guanosine nucleotide. The fluorescence change of mant-GTP upon binding to IF2 was monitored by stopped flow analysis of Fluorescence Resonance Energy Transfer (FRET) and measured after passing through a KV408 filter (Schott, Mainz, Germany). The concentration dependence of the apparent reaction rates allowed the calculation of the rate constants of mant-GTP binding to (k_+1_) and dissociation from (k_−1_) IF2 from the slope and the intercept of the plot, respectively.

### 4.6. Binding of IF2 to 50S Ribosomal Subunits 

IF2 or IF2H301 mutants (30 pmoles) were incubated 15 minutes at 37 °C with increasing amounts (0–100 pmoles) of 50S ribosomal subunits in 90 µL of Buffer A containing 150 mM NH_4_Cl. After centrifugation for 20 min at 4 °C at 100 K rpm in an ultracentrifuge (RC M120 GX, rotor S100AT3-205, Sorvall, Waltham, MA, USA), 5 μL aliquots of post-ribosomal supernatant were withdrawn and loaded on SDS-(9%) PAGE. The gels were silver-stained [62], dried and the amount of IF2 was quantified by densitometry (BIO-RAD Imaging System, Milano, Italy). 

### 4.7. IF2-dependent fMet-tRNA Binding to 30S Subunits (30S IC Formation)

The reactions were performed essentially as described [60]. The samples (40 µL) were prepared in Buffer A containing 1 mM GTP (unless otherwise specified), 0.3 µM 30S, 0.45 µM each of f[^3^H]Met-tRNA, IF1 and IF3, 0.9 µM 022mRNA and increasing amounts of wtIF2 or IF2 mutants (0.1, 0.2, 0.3 and 0.4 µM). After 15 min incubation at 37 °C, the amount of 30S-bound fMet-tRNA was determined by Millipore filtration [60].

### 4.8. fMet-puromycin and Initiation Dipeptide Formation 

These tests were carried out essentially as described [60]. The puromycin reaction was started by the addition of puromycin (2 mM final concentration) and 50S subunits (0.3 µM final concentration) to 30S initiation complexes, formed as described above, and was stopped by the addition of 500 µL of 1M (NH_4_)HCO_3_ pH 9.0. The f[^35^S]Met-puromycin present in each sample was extracted with 1 mL of ethyl acetate and the radioactivity present in 500 µL of the ethyl acetate phase was determined by liquid scintillation counting. To assay the initiation dipeptide formation an EF-Tu•GTP• [^14^C]Phe-tRNA ternary complex was first prepared in Buffer A incubating EF-Tu (0.3 µM final concentration) with 1 mM GTP, 3 mM phosphoenol pyruvate and pyruvate kinase (0.25 µg/mL) for 15 min at 37 °C before addition of [^14^C]Phe-tRNA (0.15 µM final concentration) and an additional incubation for 1 min at 37 °C. The actual test was then performed by mixing 30S IC (40 µL), prepared as described above, with an equal volume ternary complex to which 50S subunits were added (0.3 µM final concentration). After incubation at 37 °C for 15 min the reaction was quenched with an equal volume of 0.5 M KOH, neutralized with acetic acid and centrifuged for 5 min at 12K rpm. The dipeptides formed were analyzed by HPLC on a reversed phase column (LiChrosorb RP-8, 5 µM – Merck, Darmstadt, Germany) with an acetonitrile gradient (0–65%) in the presence of 0.1% TFA. 

### 4.9. Dissociation of IF2 from the Ribosome

All samples contained wtIF2 or IF2H301 mutants (45 pmol) in 90 µL Buffer A with 150 mM NH_4_Cl and 1 mM GTP. The initial complexes were made by incubating the individual IF2 variants with 30S ribosomal subunits (56 pmol), IF1 and IF3 (45 pmol each); the second complex was formed by subsequent addition of 50S subunits (75 pmol) to the initial complex; the third complex, consisting of a 30S IC, was prepared by incubation of IF2 with 30S subunits (56 pmol), 022 mRNA (100 pmol), IF1, IF3 and fMet-tRNA (45 pmol each); finally, a 70S IC complex was prepared by addition of 50S subunits (75 pmol) to the 30S IC described above. Prior to centrifugation at 4 °C in a Sorvall ultracentrifuge (RC M120 GX, rotor S100AT3-205) for 20 min at 100 K rpm all the complexes were incubated for 15 min at 45 °C. The dissociation of IF2 from the different ribosomal complexes was quantified as the amount of IF2 present in 5 µL of the supernatant obtained after centrifugation of the complexes. The analysis was performed on SDS-(9%) PAGE.

## Figures and Tables

**Table 1:** Effect of the IF2H301 mutations on the Kd’s of the IF2-mantGTP complexes.

Type of IF2	K_d_, µM
wild-type	10 ± 0.7
H301S	19.1 ± 0.8
H301Q	18 ± 0.9
H301R	24.7 ± 1
H301L	23.4 ± 1
H301Y	22 ± 0.9

**Table 2:** Disparate phenotypes of IF2H301 mutants.

Activity	IF2wt	H301Y	H301Q	H301R	H301S	H301L
GTP binding	+++	++	++	++	++	++
GTP hydrolysis	+++	−	+++	−	−	−
30S IC formation	+++	+++	+++	+++	+++	+++
fMet-puromycin formation	+++	+	+++	++	+	+
Initiation dipeptide (fMet-Phe) formation	+++	−	+	−	−	−
Affinity for 70S	+	+++	+++	+	++	++
Affinity for 30S IC	+++	++	++	+	++	++
Dissociation from 70S IC	+	−	−	−	−	−
Dominant lethal phenotype	−	+	−	−	+	+

The strength of each activity is indicated in decreasing order from +++ to −.

## Supplementary Materials

Supplementary materials can be found at https://www.mdpi.com/1422-0067/21/3/735/s1.

## Abbreviations

IF	(translation) initiation factor
GDP	guanosine diphosphate
GTP	guanosine triphosphate
ppGpp	guanosine tetraphosphate
SDS	sodium dodecyl sulfate
PAGE	polyacrylamide electrophoresis
30S IC	30S initiation complex
70S IC	70S initiation complex
DTT	dithiothreitol
PMSF	phenylmethylsulfonyl fluoride
