# Supplementary material for: Disparate Phenotypes Resulting from Mutations of a Single Histidine in Switch II of Geobacillus stearothermophilus Translation Initiation Factor IF2
Source: Int J Mol Sci. 2020 Jan 22;21(3):735. doi: 10.3390/ijms21030735 (PMC7037019; doi:10.3390/ijms21030735)
Supplement: Supplementary file 1 [file ijms-21-00735-s001.pdf]

Supplementary Material

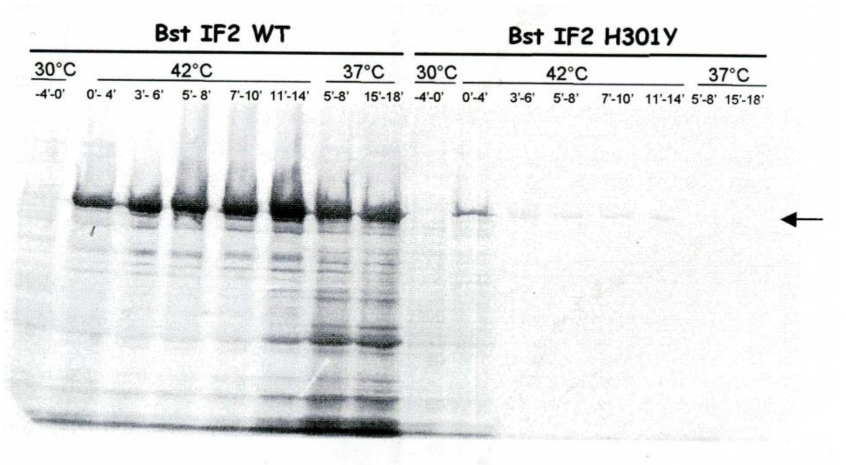

Figure S1. Pulse-chase analysis of the proteins synthesized *in vivo* following the induction of wtIF2 (left) and IF2H301Y mutant (right). Cultures of *E. coli* UT5600 cells carrying pIM401 and pIM505 were grown in M9 minimal medium at 30°C. When the cultures reached $A_{600} \cong 0.5$, eight aliquots (750 µl each) were collected and incubated at 30°C in 8 tubes. The cultures were then shifted to 45°C to induce IF2 expression and pulsed for 3 min with 2.25 µCi of [^{35}S]methionine added for the indicated times after induction. Each pulse was followed by a chase with 90 µl of 250 mM non-radioactive methionine after which the cells were incubated for 30 sec before being transferred to an ice bath After centrifugation (5 min at 13K rpm), the cells were resuspended in 0.9% NaCl (w/v), centrifuged again and then dissolved in SDS loading buffer containing 1% β-mercaptoethanol and subjected to SDS-PAGE (7.5% acrylamide). After drying, the gel was subjected to autoradiography. The arrow on the right side indicates the position of *G. stearothermophilus* IF2

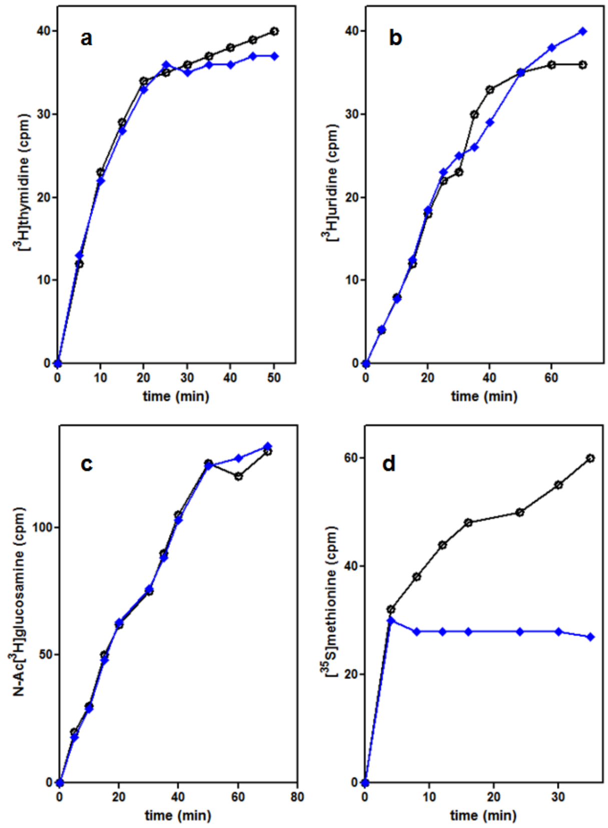

Figure S2. Effect of wtIF2 and IF2His301Y overproduction on *in vivo* macromolecular synthesis. The precursors (a) [^{3}H]thymidine; (b) [^{3}H] uridine; (c) N-Ac[^{3}H] glucosamine and (d) [^{35}S] methionine were added at time 0 to cultures of *E. coli* cells harbouring plasmids expressing either wtIF2 ( ) or IF2H301Y( ) and simultaneously transferred to 42°C for 20 min before being incubated at 30°C for the indicated times.

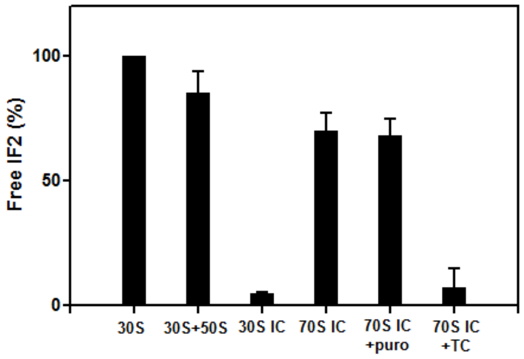

Figure S3. Dissociation of wtIF2 from the ribosomes. The histogram bars represent the amount of free (i.e. not ribosome-bound) wtIF2 present in the post-ribosomal supernatant after ultracentrifugation of samples containing 1 mM GTP and as indicated: wtIF2 and 30S ribosomal subunits; wtIF2, 30S and 50S ribosomal subunits; wtIF2 and all the components necessary for the formation of a 30S IC; wtIF2, 30S IC and 50S subunit forming a 70S IC; 70S IC and puromycin; 70S IC and a ternary complex consisting of EF-Tu, GTP and Phe-tRNA. The amounts of free IF2 detected in the samples containing only 30S subunits are taken as equal to 100% and the amounts of free IF2 detected in the other samples are expressed as % of this amount. More experimental details are given in Materials and Methods of the main text.

Materials and Methods

Strains and growth media *E. coli* strain UT5600 carrying λcI^{ts} repressor (expressed by plasmid pcI*857*) and *G. stearothermophilus infB* in plasmid pPlc2833 carrying the *G. stearothermophilus infB* gene behind the lambda pL promoter [**1**] (a vector which we named pIM401[2]) or the newly prepared plasmids (pIM500 series) carrying His301 mutants of *infB* were grown either in LB medium (1% Bacto-tryptone, 0.5% yeast extract, 0.5% NaCl) or in M9 minimal medium supplemented with 0.05% casaminoacids, 0.005% vitamin B_1, 2 mM $MgSO_4$ and an amino acid mixture containing 0.08 mg/ml of proline, leucine and tryptophan. In both cases, the medium was supplemented with kanamycin (25 μg/μl) and ampicillin (60 μg/μl).
E. coli JM109 [**3**] was used as a recipient for construction of the shuttle vector carrying *infB* gene (wild-type and mutant alleles) which was subsequently transferred to a *Saccharomyces cerevisiae* expression system.
Saccharomyces cerevisiae expression system
S. cerevisiae strain S150-PA1 (MAT*a leu2,3-112 trp1-289 his3-Δ1 ura3-52 lys2::TRP1::PDI*) was grown as indicated below either in YP rich medium (1% yeast extract, 2% peptone, 0.3% KH_2PO_4) supplemented with 2% glucose (named YPD) or in minimal medium (0.67% yeast nitrogen base w/o amino acids (Difco), 2% glucose, supplemented with 0.005% of lysine, histidine and leucine).

The overexpression of *G. stearothermophilus* IF2 mutants cloned under the control of a β-galactose inducible promoter was achieved by addition of GALYP medium (YP medium containing 4% galactose) to cells growing in minimal medium.

<u>*Site directed mutagenesis*</u> For the substitution of His301 in *G. stearothermophilus* IF2, the *Eco*RI/*Pvu*II fragment was excised from pIM401and subcloned in pSELECT (Promega) digested with *Eco*RI/*Sma*I, and subjected to site-directed mutagenesis (Altered Sites Mutagenesis System, Promega) using the following mutagenic oligonucleotides: a) 5' ACA CCA GGC **AGT** GAA GCG TT 3'; b) 5' CAC CAG GC**C AG**G AAG CG 3'; c) 5' ACA CCA GGC **CGT** GAA GCG TT 3'; d) 5' ACA CCA GGC **CTT** GAA GCG TT 3' and e) 5' CAC CAG GC**T AT**G AAG CG 3' for the substitutions of histidine 301 with serine, glutamine, arginine, leucine and tyrosine, respectively. The mutagenized fragments were subsequently transferred to the expression vector pIM401. Using this procedure 5 different plasmids were obtained:

pIM501 *infBH301S*
pIM502 *infBH301Q*
pIM503 *infBH301R*
pIM504 *infBH301L*
pIM505 *infBH301Y*

<u>*Expression of G. stearothermophilus wtIF2 and IF2H301 mutants*</u> The expression system in *E. coli* UT5600 carrying the pIM40 (wtIF2), pIM502 (H301Q) or pIM503 (H301R) was the same as previously described [2]. Upon growth at 30°C in LB medium containing ampicillin (60 μg/ml) and kanamycin (25 μg/ml) to an A_{600} of about 0.9, overexpression of IF2 was obtained following 20 min of induction at 42°C and further incubation at 37°C for 1 h. The cells were then harvested, resuspended in 20 mM Tris-HCl, pH 7.1 buffer containing 60 mM NH_4Cl, 10 mM $MgAc_2$, 0.2 mM phenylmethylsulfonyl fluoride (PMSF) and rapidly frozen at -80°C.

<u>*Purification of G. stearothermophilus wt IF2 and IF2 mutants*</u> Approximately 5 gr of *E. coli* UT5600 cells were resuspended in 20 mM Tris-HCl, pH 7.1 buffer containing 60 mM NH_4Cl, 10 mM $MgAc_2$, 0.2 mM phenylmethylsulfonyl fluoride (PMSF), 5 mM β-mercaptoethanol and supplemented with DNase I (2.5 μg/g of cells) and sonicated in an ice bath. The supernatant resulting after centrifugation for 1 h at 30K x g in rotor SA-600 (Sorvall), was centrifuged at 100K x g in Type 50Ti rotor (Beckman) in a Kontron ultracentrifuge in the presence of 1 M NH_4Cl. The resulting supernatant (S100) was diluted 15 times in 20 mM Tris-HCl, pH 7.1 buffer containing 0.1 mM EDTA, 5% glycerol, 5 mM β-mercaptoethanol, 0.2 mM PMSF and loaded onto a phosphocellulose (Whatman P-11) column (10 ml of bed volume) equilibrated in the above buffer containing 50mM NH_4Cl. After washing with equilibration buffer (4 x bed volumes), elution was performed in steps corresponding to 4 bed volumes with 50 mM increments of the salt concentration from 200 mM NH_4Cl to 500 mM NH_4Cl collecting 5 ml fractions. Following SDS-polyacrylamide gel electrophoresis (SDS-PAGE) analysis, the fractions containing IF2 were pooled, diluted with 20 mM Tris-HCl, pH 7.1 buffer containing 0.1 mM EDTA, 10% glycerol, 1 mM DTT and loaded onto a DEAE-cellulose (Whatmann DE-52) column (12 ml of bed volume) equilibrated with the same buffer containing 50 mM NH_4Cl. After washing with equilibration buffer (4 bed volumes), 20 mM Tris-HCl, pH 7.1, 0.1 mM EDTA, 10% glycerol, 1mM DTT elution was performed with a linear NH_4Cl gradient (50- 450 mM) in the same buffer. Following SDS-PAGE analysis, the fractions containing IF2 were pooled, dialyzed against 20mM Tris-HCl, pH 7.1 buffer containing 0.1mM EDTA, 5% glycerol, 5 mM β-mercaptoethanol, 0.2 mM PMSF and 200 mM NH_4Cl and concentrated using Centrex UF-2 10kD (Schleicher & Schuell).

<u>*Construction of the expression vector for S. cerevisiae containing G. stearothermophilus IF2 mutants*</u>

To construct the plasmid for the heterologous expression of the toxic IF2H301 mutants, the shuttle vector pEMBLyex2 [4] was used. The IF2H301 mutated genes were cloned into the unique BamH1 site immediately after the inducible galactose promoter.

Transformation of S. cerevisiae with the shuttle vector

S. cerevisiae S150-PA1 was transformed using the LiCl method previously described [5]. The cells were grown in YPD medium at 30°C to $A_{640} \cong 0.5$, harvested by centrifugation for 7 minutes at 1,100 x g, resuspended in 1/5 volume of TE (10mM Tris-HCl, pH 8.0 and 1mM EDTA)and centrifuged again. The pellet was then resuspended in in TE containing 0.1mM LiCl (1/30 of starting volume) and left on ice for 3 to 8 hours. Approx. 20 µl of DNA solution (≥ 5µg of plasmid DNA) were added to 200 µl competent cells which were then incubated at room temperature for 30 minutes. After addition of 1.5ml of 40% PEG 4000 in 0.1M LiCl in TE, the solution was gently mixed and kept for 1 h at room temperature. The cells were then heat shocked at 42°C for 15 min and centrifuged at 500 x g in SA600 rotor (Sorvall). The cells pellet were washed with 2 ml of sterile water to remove PEG (without resuspending the cells). After this step, the cells were resuspended in 200 µl of water and transformants were selected on minimal medium plates containing histidine, leucine and lysine.

Expression and purification of G. stearothermophilus IF2 from S. cerevisiae

The expression system in *S. cerevisiae* carrying pEMBLyex2 + *G. stearothermophilus infB* (wild-type and mutants) was the same as that previously described [4] for the pEMBLyex2 derivative (i.e. YEpsec1). Upon growth at 30°C in minimal medium (described above) to an A_{640} of about 4 - 5, overexpression was obtained by addition of 3 volumes of GALYP medium and incubation of the cultures for 5 days in a shaking water bath at 30°C.

For the purification of IF2 from *S. cerevisiae* cells, around 30 g of cells were ground in a mortar with Glass beads (Sigma 425-600 microns) 1 g/g of cells. The cell paste was homogenized with 30 ml of ice-cold 10 mM Tris-HCl, pH 7.7 buffer containing 60 mM NH_4Cl, 5 mM $MgAc_2$, 5 mM β-mercaptoethanol and 0.2 mM PMSF. The supernatant resulting after centrifugation for 1 h at 30,000 x g in rotor SA-600 (Sorvall) was centrifuged at 100K x g in Type 50Ti rotor (Beckman) in the presence of 1 M NH_4Cl.

References

1. Brombach, M., Gualerzi, C.O., Nakamura, Y., Pon, C.L. (1986) Molecular cloning and sequence of the *Bacillus stearothermophilus* translational initiation factor IF2 gene. Mol. Gen. Genet. 205:97-1022.
2. Spurio, R., Severini. M., La Teana, A., Canonaco, M.A., Pawlik, R.T., Gualerzi, C.O., Pon, C.L. (1993) Novel structural and functional aspects of translational initiation factor IF2. In The Translational Apparatus (Nierhaus K.H. et al., eds) Plenum Press, New York. p. 241-252.
3. Hanahan, D. (1985) in DNA Cloning 1 - A Practical Approach Core Techniques 2nd Edition. Glover, D.M., Hames, B.D. Eds. IRL Press Ltd, UK pp.109-135.
4. Baldari C., Murray, J.A., Ghiara, P., Cesareni, G., Galeotti, C.L .(1987) A novel leader peptide which allows efficient secretion of a fragment of human interleukin 1 beta in *Saccharomyces cerevisiae* EMBO J. 6:229-234.
5. Ito, H., Fukuda, Y., Murata, K., Kimura, A. (1983) Transformation of intact yeast cells treated with alkali cations. J. Bacteriol. 153:163-168.
